# Identification of autophagy-related risk signatures for the prognosis, diagnosis, and targeted therapy in cervical cancer

**DOI:** 10.1186/s12935-021-02073-w

**Published:** 2021-07-08

**Authors:** Dan Meng, Hua Jin, Xing Zhang, Wenjing Yan, Qianqian Xia, Siyuan Shen, Shuqian Xie, Mengjing Cui, Bo Ding, Yun Gu, Shizhi Wang

**Affiliations:** 1 Key Laboratory of Environmental Medicine Engineering, Ministry of Education, School of Public Health, Southeast University, 87 Dingjiaqiao, Gulou District, Nanjing, 210009 China; 2 Clinical Laboratory, Affiliated Tumor Hospital of Nantong University (Nantong Tumor Hospital), Nantong, China; 3 Department of Gynecologic Oncology, Affiliated Cancer Hospital of Nanjing Medical University, Jiangsu Cancer Hospital, Nanjing, China; 4 Department of Pathology, Women’s Hospital of Nanjing Medical University, Nanjing Maternity and Child Health Care Hospital, No.123, Tianfei Alley, Mochou Road, Nanjing, 210004 China

**Keywords:** Cervical cancer, Autophagy, mRNA, Biomarkers, qPCR

## Abstract

**Background:**

To rummage autophagy-related prognostic, diagnostic, and therapeutic biomarkers in cervical cancer (CC).

**Methods:**

The RNA-sequence and clinical information were from the TCGA and GTEx databases. We operated Cox regression to determine signatures related to overall survival (OS) and recurrence-free survival (RFS) respectively. The diagnostic and therapeutic effectiveness of prognostic biomarkers were further explored.

**Results:**

We identified nine (*VAMP7*, *MTMR14*, *ATG4D*, *KLHL24*, *TP73*, *NAMPT*, *CD46*, *HGS*, *ATG4C*) and three risk signatures (*SERPINA1*, *HSPB8*, *SUPT20H*) with prognostic values for OS and RFS respectively. Six risk signatures (*ATG4C*, *ATG4D*, *CD46*, *TP73*, *SERPINA1*, *HSPB8*) were selected for qPCR. We screened five prognostic signatures(*ATG4C*, *CD46*, *HSPB8*, *MTMR14*, *NAMPT*) with diagnostic function through the GEO database. Correlation between our models and treatment targets certificated the prognostic score provided a reference for precision medicine.

**Conclusions:**

We constructed OS and RFS prognostic models in CC. Autophagy-related risk signatures might serve as diagnostic and therapeutic biomarkers.

**Supplementary Information:**

The online version contains supplementary material available at 10.1186/s12935-021-02073-w.

## Background

Cervical cancer (CC) is one of the most common gynecological malignancies in women [1]. It is estimated that 600,000 women are diagnosed as CC, and more than 340,000 people die from CC each year. The continuous infection of high risk human papillomavirus (HR-HPV) is a necessary condition for CC, but its specific mechanism and co-factors have not yet been clarified [2]. Studies have shown that more than 70% of CC patients are diagnosed as advanced, and 15% to 61% of patients have lymph node or distant metastasis and recurrence, then the survival rate of these patients is significantly reduced [3]. Although the use of HPV vaccine and the progress of surgery, radiotherapy and chemotherapy have reduced the incidence and mortality of CC, late metastasis, recurrence and drug resistance are still huge challenges for its clinical treatment [4]. Limited by the economic level, the burden of disease is heavier in areas with scarce medical resources [5]. To reduce the burden and provide better treatment to patients, it is necessary to make accurate judgments on the prognosis of patients in order to achieve the purpose of formulating reasonable treatment plans and avoiding overtreatment or undertreatment. In recent years, autophagy has been discovered to play a momentous role in CC [6, 7].

Autophagy is an evolutionarily conserved important process in eukaryotes, which is responsible for the turnover of intracellular substances [8–10]. It has not been fully clarified whether autophagy plays a positive or negative role in physiological or pathological processes including cancer. Studies have shown that autophagy dysregulation plays a vital role in the occurrence and development of CC. Hu et al*.* found that autophagy was undermined in CC cells with gradually reduced positive expression rate of autophagy-related proteins Beclin1 and LC3 in the normal cervical group, CIN (cervical intraepithelial neoplasia) III group, and CC group [11]. Li et al*.* discovered that high-risk HPV infection could promote the development of CC by inhibiting autophagy of the host [12]. Zhang et al*.* reported that microtubule-associated protein 7 (MAP7) promoted the migration, invasion and progression of CC by regulating the autophagy pathway [13].

RNA sequence (RNA-seq) is a technique for observing the differences of cells at the gene level, thereby analyzing the biological behavior of different cells. At present, through the construction and verification of prediction models, a large number of studies have applied RNA-seq results to the diagnosis, treatment, and prognosis of CC. Wu et al*.* forecasted two lncRNA signatures as potential prognostic biomarkers in CC based on the TCGA database [14]. Ding et al*.* screened immune-related genes’ (IRGs) prognostic implications in cervical cancer and endometrial cancer [15]. Wang et al. identified *DAPK1* promoter hypermethylation as a biomarker for intraepithelial lesion and cervical cancer [16]. However, the literature on the prognostic, diagnostic and therapeutic value of autophagy-related genes in CC is scarce.

In the present study, we acquired the transcriptome profile and clinical information of CC patients from The Cancer Genome Atlas (TCGA) and Genotype-Tissue Expression (GTEx). According to the Human Autophagy-dedicated Database(HADb), we searched for autophagy-related genes (ARGs) in CC. After Cox regression analysis, ARGs related to overall survival (OS) and recurrence-free survival (RFS) were determined and their respective prognostic models were established. Kaplan–Meier curve, area under the curve (AUC) and nomogram all indicated that these models had an excellent predictive performance. Endometrial cancer (UCEC) and head and neck cancer (HNSCC) were used as external verification data to verify the effectiveness of the models. The functions of ARGs included in the prognostic models were further analyzed by Gene Set Enrichment Analysis (GSEA). Finally, the diagnostic value of risk signatures was detected based on the Gene Expression Omnibus (GEO) database. We found that the risk score was significantly correlated with clinical treatment targets. In this paper, we screened out valuable biomarkers for CC by constructing prognostic models containing ARGs and studied their diagnostic value and treatment orientation for clinical.

## Materials and methods

### Data gathered and pre-neatening of the training set

The RNA-seq transcriptome data (HTSeq-Counts) composed of 306 CC with clinical information and 3 normal samples was downloaded from the TCGA data portal (https://tcga-data.nci.nih.gov/tcga/). Five normal cervix samples from the GTEx portal (http://gtexportal.org) were added to expand the number of normal samples. For subsequent difference analysis, we normalized the transcriptome data from these two datasets and integrated data by R program (http://www.r-project.org/webcite) v4.0.2 using the limma package (http://www.bioconductor.org/). HADb (http://www.autophagy.lu/) is the first Human Autophagy-appropriated Database. We obtained a list of 232 human autophagy genes, as shown in Additional file 1. Subsequently, 217 autophagy-related genes (ARGs) expressed in CC were shown in Additional file 2. We matched the autophagy-related gene expression profiles with the OS and RFS according to the patient’s ID numbers respectively, and those that did not match were excluded.

### Identification and functional annotation of the differently expressed autophagy-related genes (DEARGs)

DEARGs were appraised by the R program using the edgeR package (Bioconductor-edgeR) with cut-off values as FDR < 0.05 and |log_2_FC|> 1. To investigate the biomedical molecular mechanism of DEARGs, we carried out Gene Ontology (GO) annotation and Kyoto Encyclopedia of Genes and Genomes (KEGG) pathway enrichment under R environment using clusterProfiler package(Bioconductor-clusterProfiler). Then the STRING database (https://string-db.org/) was exploited to construct protein–protein interaction (PPI) network of DEARGs, and we imported the data into the software of Cytoscape 3.7.2 to visualize the interaction of PPI network.

### Construction of the ARGs-based risk signatures

Firstly, univariate Cox regression analysis was performed to screen ARGs related to OS and RFS, and *P* < 0.05 was considered significant. Subsequently, to prevent overfitting of the models, we conducted Least absolute shrinkage and selection operator (Lasso) regression by using the R package “glmnet” (https://CRAN.R-project.org/package=glmnet). Finally, multivariate Cox regression analysis was carried out to evaluate the risk signatures of the predictive model and obtain their regression coefficients. We calculated the patient's risk score based on the expression level of the prognostic genes and the risk coefficient. The risk score was used to predict the prognosis of CC patients, and then patients were divided into high- and low-risk groups according to the median risk score (*P* < 0.05). The Kaplan–Meier (K–M) survival curve was plotted to evaluate the statistical differences in OS and RFS between the two groups respectively. In addition, we generated receiver operating characteristic (ROC) curves to determine the accuracy of the prognostic models.

Univariate and multivariate Cox regression analyses were conducted to investigate whether autophagy-related risk biomarkers could be regarded as independent predictors of OS and RFS in CC patients from the TCGA data set. Clinical parameters such as age, stage, grade, T, race and BMI were considered for OS. The clinical information related to RFS was incomplete, we only included age, stage, and grade for independent prognostic analysis. The nomograms were generated to visualize the risk score and the survival probability of the patient calculated according to the prognostic genes in the OS and RFS model using the “rms” and “survival” packages, thus realizing the combination of bioinformatics research and clinical practice. The calibration curve of the prediction model was used to evaluate the difference between the predicted probability and the actual probability.

### Validation of the prognostic model in the testing set

To verify the interpretability and prediction accuracy of the model, we introduced two other cancer types, they were endometrial cancer (UCEC) and head and neck cancer (HNSCC), both of which were directly or indirectly related to CC. HPV infection is an important risk factor for head and neck cancer [17, 18]. Endometrial cancer is also a common uterine malignant tumor. It has been reported that CC may cause endometrial cancer after radiotherapy [19].

The mRNA expression data and clinical OS data of patients with UCEC were from UCSC Xena (http://xena.ucsc.edu/). GSE117973, a global gene expression analysis of 77 primary head and neck cancer cases consisting of clinical progression-free survival (PFS) information, was downloaded from the comprehensive Gene Expression Omnibus (GEO; https://www.ncbi.nlm.nih.gov/geo/). Similarly, we calculated the patient's risk score based on the risk signatures selected in the training set and divided samples into high- and low-risk groups, the K-M survival curves and ROC curves were plotted to verify the validity of the models in the testing set.

### Detection of the diagnostic values of risk indicators in GEO database

The diagnostic values of the risk indicators for OS and RFS were assessed using ROC curve analysis from consolidated data sets from GSE63514 (24 normal samples and 28 CC), GSE75132 (21 normal samples and 20 CC), GSE6791 (8 normal samples and 20 CC). We combined the data and removed the batch effect for further analysis.

### Gene Set Enrichment Analysis of the 12 risk signatures

GSEA 4.1.0 was used to demonstrate the role of each gene in CC. We chose C2.CP: KEGG.V7.0.symbols.gmt file as the reference gene set file, the values of *P* < 0.05 and FDR < 0.25 after performing 1,000 permutations were considered a significant difference.

### Correlation between risk score and clinical therapeutic targets

Here we studied the relationship between risk score and the common targeted therapeutic targets in CC by Pearson’s correlation analysis. The therapy targets are listed as follow: AKT serine/threonine kinase 1 (*AKT1*, *AKT*), BCL2 apoptosis regulator (*BCL2*,* Bcl-2*, *PPP1R50*), mechanistic target of rapamycin kinase (*MTOR*, *FRAP*), tumor protein p53 (*TP53*, *P53*), vascular endothelial growth factor A (*VEGFA*, *VEGF*).

### Expression of the 12 risk signatures at the protein level

The Human Protein Atlas database(https://www.proteinatlas.org/) is a free data resource that contains more than 26,000 kinds of antibodies. The immunohistochemical results of 12 risk signatures in normal and pathological tissues were found in this database.

### RNA expression detection of selected risk signatures in cervical cancer and normal tissue samples

To detect certain risk signatures(*ATG4C*, *ATG4D*, *CD46*, *TP73*, *SERPINA1*, *HSPB8*) expression in human samples by qPCR, a total of seven fresh CC tissues and paired adjacent non-tumor tissues were acquired from patients between September 2019 and October 2020 at Zhongda Hospital. RNA extraction and qRT-PCR can be described in the previous literature published by our research group [20]. The primer sequences involved in this study are shown in Additional file 3. All samples were stored at − 80 °C before total RNA extraction.

### Statistical analysis

Statistical analyses were performed with the use of R 4.0.2 (www.r-project.org), SPSS 26, and GraphPad Prism 8.0.2. All statistical tests were two-sided, and *P* < 0.05 were considered statistically significant.

## Results

### Identification and functional annotation of DEARGs

**Fig. 1:**
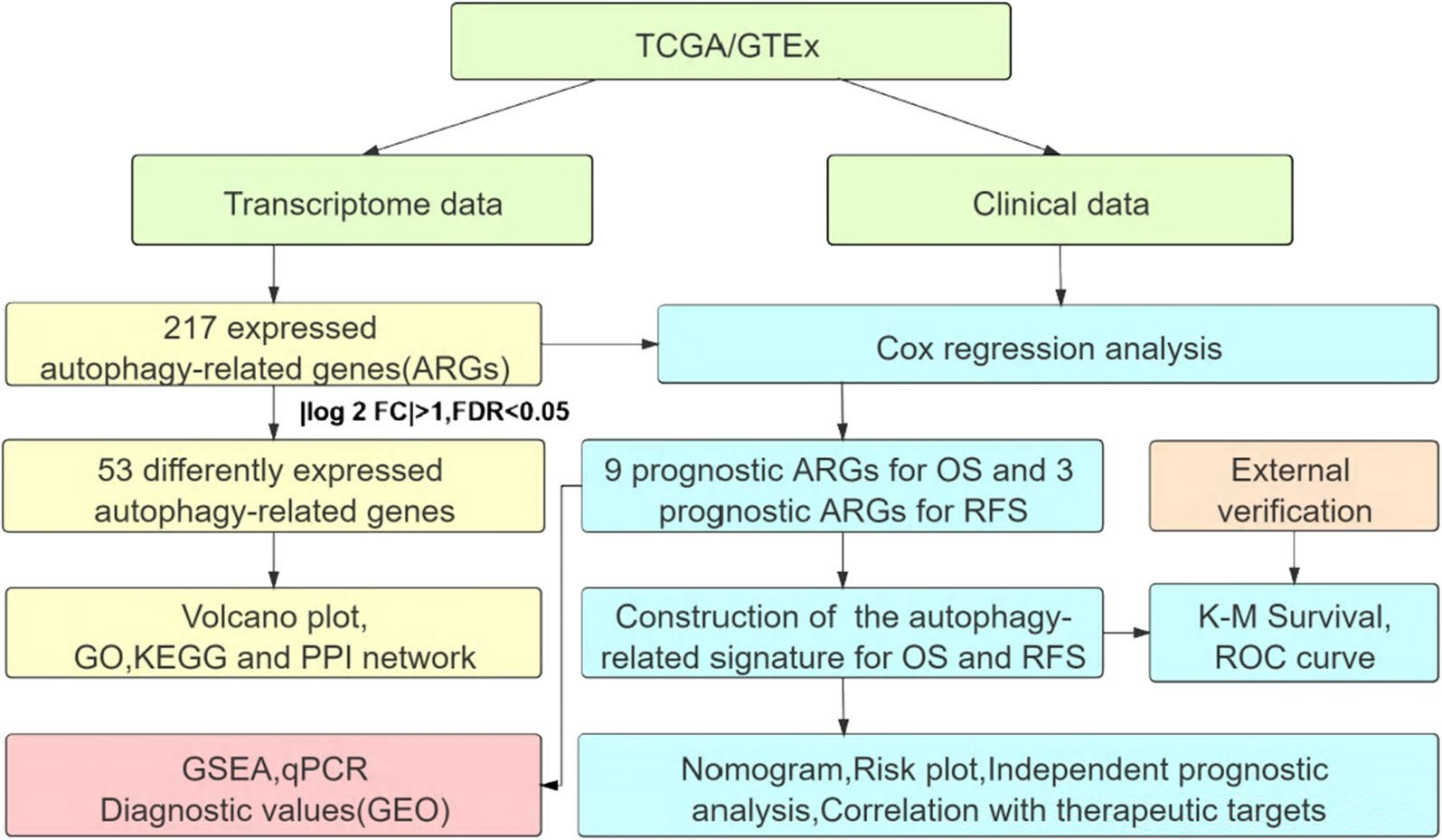
The flow diagram of the integral analysis

**Fig. 2:**
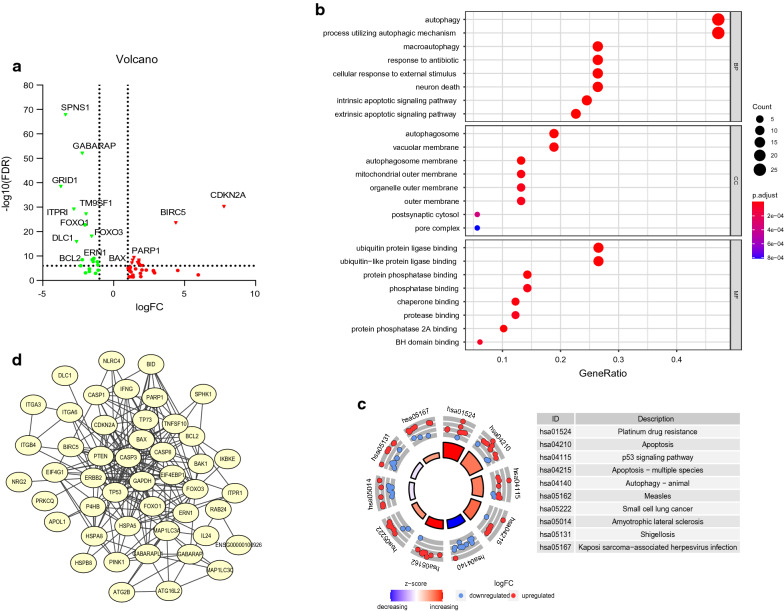
Identification and functional annotation of differently expressed autophagy-related genes (DEARGs) in CC patients. **a** Volcano plot for DEARGs in the tumor and normal samples, in the volcano map, we used the ordinate “6” as the boundary for gene annotation. **b** Top 8 GO analysis of 53 DEARGs from the three aspects of BP, CC and MF. **c** Top 10 significant KEGG signal pathways. **d** PPI network of 53 DEARGs

The flow diagram of the integral analysis is described in Fig. 1. We obtained 217 autophagy genes expressed in 306 CC patients and 8 normal controls. Among these expressed autophagy genes, there were 53 differentially expressed genes (Fig. 2a), including 21 down-regulated and 32 up-regulated (FDR < 0.05, |log_2_FC|> 1). The box plot revealed the expression pattern of differential genes in tumor and normal samples (Additional file 7: Figure S1a), all DEARGs were represented in Additional file 4. To rummage the signal pathways of DEARGs, we performed GO and KEGG enrichment analysis (Fig. 2b, c). In the term of biological process (BP), the enriched pathways such as autophagy, macroautophagy, cellular response to external stimulus, intrinsic apoptotic signaling pathway and extrinsic apoptotic signaling pathway closely related to cell proliferation and migration. For the cellular component (CC) part, autophagosome, autophagosome membrane and vacuolar membrane were involved in autophagy. The results of molecular function (MF) of the genes showed these genes participated in many binding reactions. KEGG analysis showed 53 DEARGs were included in critical pathways associated with cancer development, such as apoptosis, autophagy—animal, platinum drug resistance and p53 signaling pathway. The detailed results of significant top 8 GO annotation and top 10 KEGG pathway enrichment were shown in Additional file 5. The PPI network showed the connection between the various genes (Fig. 2d). The hub genes might be identified by the number of connections, CASP3 had the most connected nodes with other genes (Additional file 7: Figure S1b).

### Construction and verification of autophagy prognostic model in the training set

**Fig. 3:**
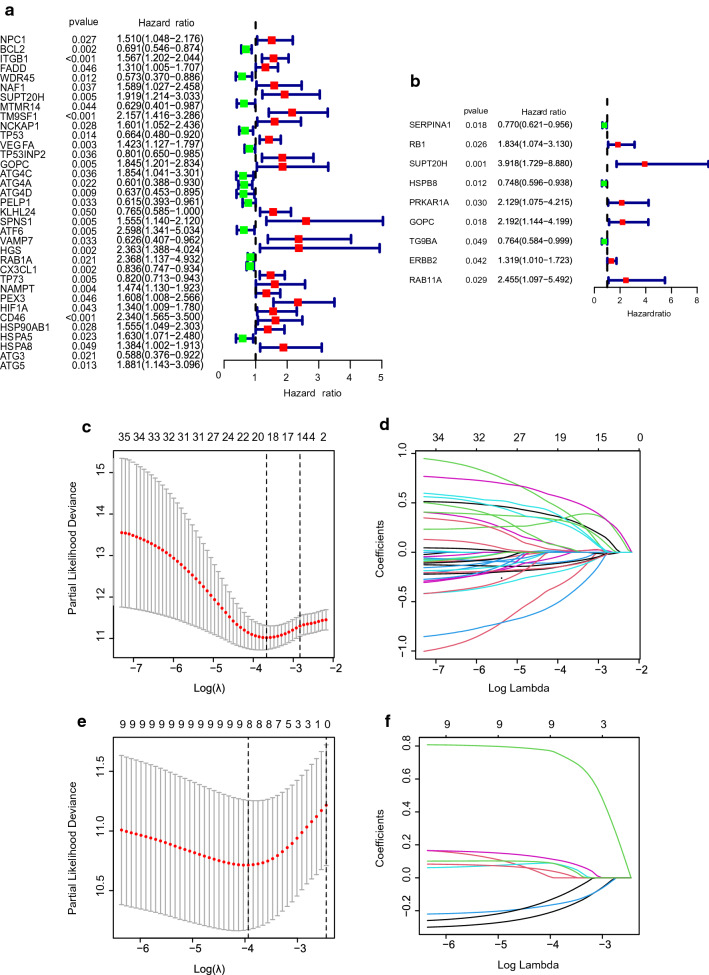
Construction of OS and RFS risk prognostic models. **a** Univariate Cox regression analysis of the risk signatures for OS. **b** Univariate Cox regression analysis of the risk signatures for RFS. **c**, **d** Lasso Cox regression model for OS. **e**, **f** Lasso Cox regression model for RFS

We first performed univariate Cox regression analysis to find autophagy gene signatures related to OS and RFS respectively. Among the 35 genes related to the OS, 13 genes were protective with HR < 1, and 22 genes were dangerous with HR > 1 (Fig. 3a). Among the 9 genes related to the RFS, 3 genes were protective with HR < 1, and 6 genes were dangerous with HR > 1 (Fig. 3b). Lasso regression was adopted to further screen variables to ensure the stability of our model, and finally 20 candidate genes related to OS and 8 candidate genes related to RFS were obtained (Fig. 3c–f). Multivariate Cox regression analysis was utilized to determine the genes that constructed the predictive model and their regression coefficients. For OS and RFS models, 9 autophagy-related gene signatures (*VAMP7*, *MTMR14*, *ATG4D*, *KLHL24*, *TP73*, *NAMPT*, *CD46*, *HGS*, *ATG4C*) and 3 autophagy-related gene signatures (*SERPINA1*, *HSPB8*, *SUPT20H*) were gotten. The coefficient of each gene was shown in Table 1. Moreover, we could find that at the level of mRNA or protein, not all risk signatures related to survival were differential genes (Additional file 7: Figure S2a-l). The results from the K-M analysis of single-gene illustrated 8 genes were significantly related to survival, including five protective genes like *ATG4D*, *KLHL24*, *TP73*, *HSPB8*, *SERPINA1* (*P* = 0.025, 0.009, 0.030, 0.050, 0.014 respectively) and three dangerous genes like *CD46*, *HGS*, *SUPT20H* (*P* = 9.609e−04, 0.007, 0.014 respectively), which was consistent with the Cox regression analysis (Additional file 7: Figure S3a–l). Simultaneously, it could be speculated that these risk genes might have a synergistic effect, not just individual genes. According to the expression of model genes and regression coefficients to calculate the patient’s risk score, the formula was as follows:

$$ Risk~score = \mathop \sum \limits_{{i = 1}}^{n} Coef_{i} *x_{i} $$

**Table 1:** Multivariate Cox regression analyses of OS and RFS in CC patients

Gene	Coefficient	HR (95%CI)	*P* value
OS			
*VAMP7*	− 0.99787	0.36866 (0.21117–0.64362)	0.00045
*CD46*	0.71823	2.05081 (1.29362–3.25118)	0.00225
*NAMPT*	0.41726	1.51780 (1.13852–2.02344)	0.00445
*ATG4C*	1.02923	2.79890 (1.35789–5.76911)	0.00529
*HGS*	0.75585	2.12943 (1.20152–3.77393)	0.00963
*TP73*	− 0.20373	0.81569 (0.69885–0.95205)	0.00979
*MTMR14*	− 0.64426	0.52505 (0.31495–0.87530)	0.01348
*KLHL24*	− 0.28758	0.75008 (0.56843–0.98978)	0.04209
*ATG4D*	− 0.36031	0.69746 (0.49070–0.99134)	0.04460
RFS			
*SERPINA1*	− 0.34683	0.70693 (0.55833–0.89507)	0.00397
*SUPT20H*	1.15581	3.17661 (1.47404–6.84571)	0.00317
*HSPB8*	− 0.24263	0.78457 (0.63195–0.97403)	0.02792

Among them, coef represents the coefficient and x_i_ represents the relative expression value of each risk signature.

**Fig. 4:**
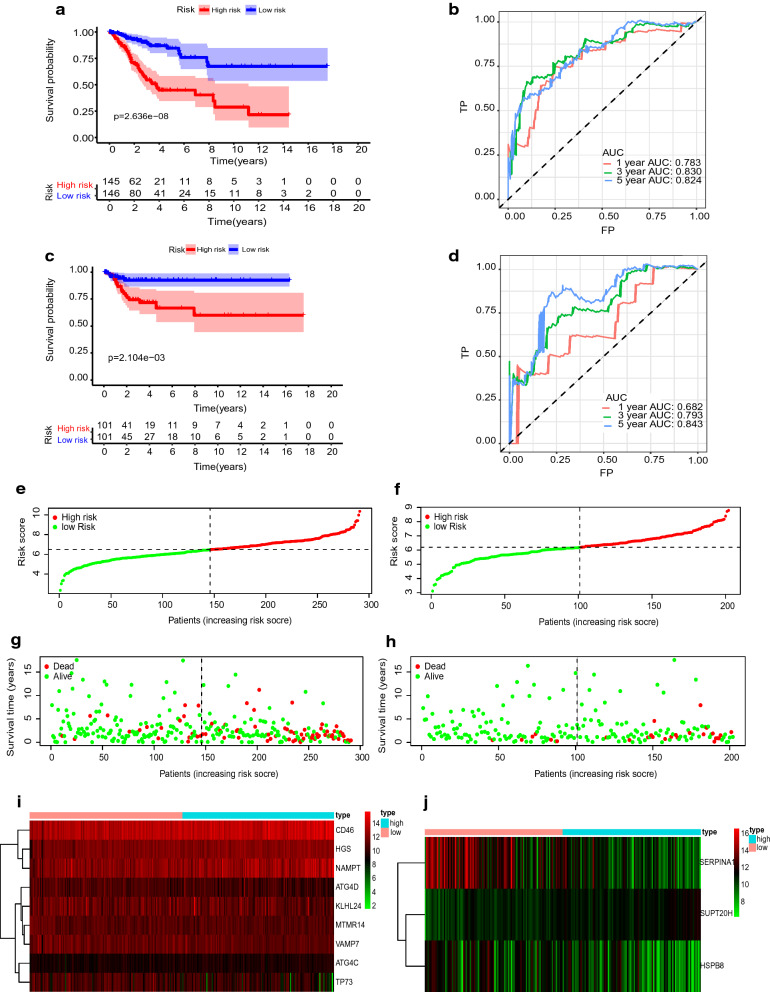
Characteristics of prognostic gene signatures. **a**, **c** K–M curves for OS (**a**) and RFS (**c**) in the high- and low-risk groups when stratified by the autophagy-related signatures. **b**, **d** ROC curve of risk score at 1,3,5 years for OS (**b**) and RFS (**d**) respectively. **e**, **f** Distribution of OS-related risk score and RFS-related risk score, the black dotted line is the optimal cut-off value for dividing patients into low- and high-risk groups. **g**, **h** Distribution of patient survival time, and status. **i**, **j** Heatmap of autophagy-related gene expression profiles in the prognostic signature of CC

The median risk score was applied as a cut-off value to divide patients into high- and low-risk groups. The OS and RFS survival analysis respectively between the two groups were significantly different (Fig. 4a, c, P = 2.636e−08 for OS, *P* = 2.104e−03 for RFS), lower risk score generally presages a better survival prognosis.

The AUC of ROC analyses at 1, 3, and 5 years was 0.783, 0.830, and 0.824 for OS and 0.682, 0.793, and 0.843 for RFS (Fig. 4b, d). Additionally, the risk score, the number of survivals and the expression of model genes between the high- and low-risk group had a difference. The expression of protective genes was higher in the low-risk group, and the expression of dangerous genes was higher in the high-risk group. The low-risk groups had fewer deaths than the high-risk groups (Fig. 4e–j).

### OS and RFS prognostic risk models were independent predictive indicators in the CC patients of the TCGA database

**Fig. 5:**
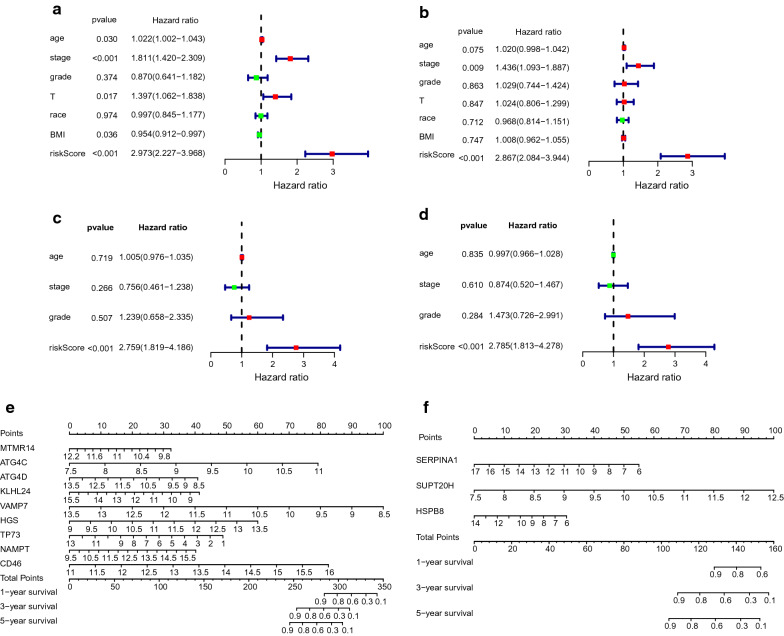
Independent prognostic analysis and nomogram diagram. **a** Univariate Cox regression analysis. Forest plot of associations between risk factors and the survival for OS. **b** Multiple Cox regression analysis. The autophagy-associated gene signature is an independent predictor of CC patients for OS. **c** Univariate Cox regression analysis. Forest plot of associations between risk factors and the survival for RFS. **d** Multiple Cox regression analysis. The autophagy-associated gene signature is an independent predictor of CC patients for RFS. **e** A nomogram of the CC cohort (training set) used to predict the OS. **f** A nomogram of the CC cohort (training set) used to predict the RFS

We further analyzed whether the risk score could assume independent predictive indicators for CC patients. The clinical information was given in Table 2. Univariate Cox regression analysis showed that age (*P* = 0.030, HR = 1.022), stage (*P* < 0.001, HR = 1.811), T (*P* = 0.017, HR = 1.397), BMI (*P* = 0.036, HR = 0.954) and the risk score (*P* < 0.001, HR = 2.973) correlated with OS of CC patients (Fig. 5a). Differently, only risk score (*P* < 0.001, HR = 2.867) was associated with RFS of CC patients (Fig. 5c). Next, we performed multivariate Cox regression analysis using the above clinical parameters and risk score. The results showed that the prognostic risk model could serve as an independent prognostic indicator to predict OS and RFS in patients respectively (*P* < 0.001, HR = 2.759 for OS, *P* < 0.001, HR = 2.785 for RFS) (Fig. 5b, d). Furthermore, multivariate Cox analysis showed that stage significantly correlated with OS (*P* = 0.009, HR = 1.436). These results demonstrated that both prognostic models could be independently used to predict OS and RFS in CC patients. The nomogram calculated the survival prediction value of the individual at 1, 3, and 5 years based on the total score and the probability of the outcome event (Fig. 5e, f). The calibration curve showed that the predicted risk was consistent with the actual risk (Additional file 7: Figure S4a–f).

**Table 2:** Clinicopathological features of cervical cancer patients

Clinical parameters	Variable	Total(253)	Percentages
OS			
Age	≤ 50	154	60.7
	> 50	99	39.3
Pathological stage	Stage I	131	51.8
	Stage II	60	23.7
	Stage III	37	14.6
	Stage IV	20	8.0
	Unknown	5	2.0
Histological grade	G1	15	5.9
	G2	117	46.2
	G3	99	39.1
	G4	1	0.4
	GX	21	8.3
T	T1	115	45.5
	T2	63	24.9
	T3	15	5.9
	T4	10	4.0
	TX	50	19.8
Race	American Indian or Alaska Native	6	2.4
	Asian	18	7.1
	Black or African American	23	9.1
	Native Hawaiian or other Pacific	1	0.4
	White	182	71.9
	Unknown	23	9.1
BMI	≤ 24	80	31.6
	> 24	173	68.4

Clinical parameters	Variable	Total (208)	Percentages
RFS			
Age	≤ 50	133	63.9
	> 50	75	36.1
Pathological stage	Stage I	113	54.3
	Stage II	50	24.0
	Stage III	34	16.3
	Stage IV	11	5.3
Histological grade	G1	17	8.2
	G2	101	48.6
	G3	89	42.8
	G4	1	0.5

### Verification of autophagy-related predictive signatures in the testing group

The effect of the prediction model is likely to be different due to changes in scenarios and populations. To verify the validity of the prognostic model and improve its generalizability, we conducted external verifications on the OS and RFS prognosis model. Since other appropriate gene expression data and clinical information about CC patients were not available, we chose UCEC and HNSCC as external verification data. We downloaded the transcriptome data of UCEC patients and clinical data including OS from the UCSC Xena database, then calculated the risk score based on the expression of nine OS model genes and regression coefficients in the UCEC patients from our testing group. Furthermore, GSE117973 containing mRNA expression information and PFS clinical data was used for validating the prognostic model of RFS and risk score was calculated. In the OS and RFS validation sets, patients were divided into high- and low-risk groups based on the calculated median risk score respectively. K–M survival curves showed that in UCEC patients and HNSCC patients, the higher risk score was associated with adverse outcomes (Additional file 7: Figure S4g, i). The AUC also proved that the risk signature had good accuracy with 0.571, 0.635, and 0.669 at 3, 5 and 7 years respectively for the OS of UCEC patients. For the PFS of HNSCC patients, the AUC was 0.443, 0.571, 0.635 at 1, 3 and 5 years respectively (Additional file 7: Figure S4h, j).

### Diagnostic value of risk signatures in GEO consolidated data sets in CC

**Fig. 6:**
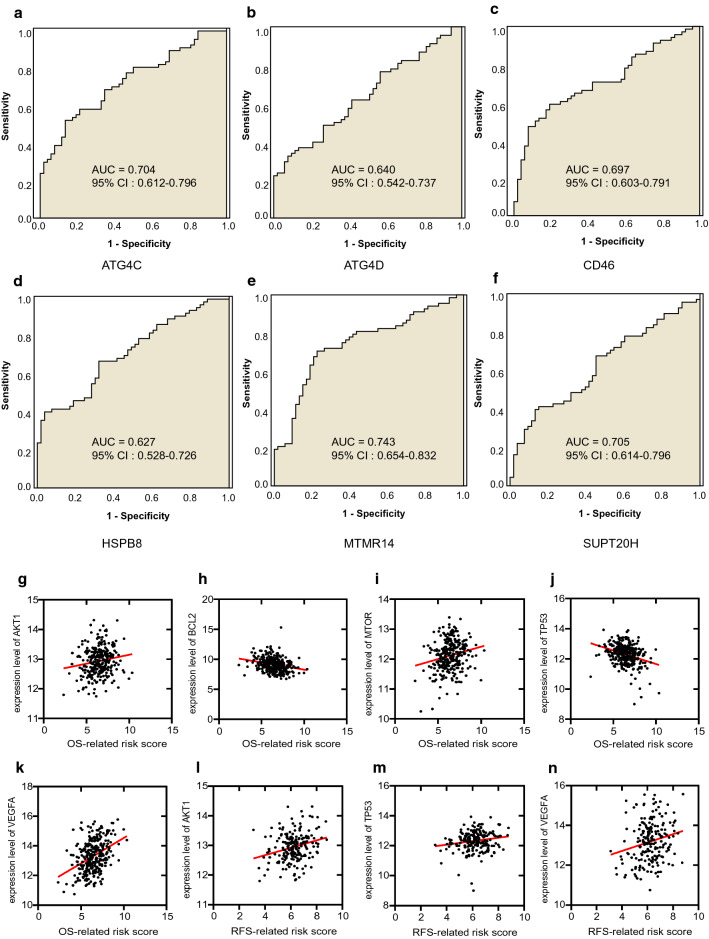
Discrimination of diagnostic value and therapeutic target sensitivity based on risk signature. **a**–**f** AUC of risk signature with diagnostic value (*ATG4C, ATG4D*, *CD46*, *HSPB8*, *MTMR14*, *SUPT20H*). **g**–**k** Correlation of the OS-related risk score and expression level of targets of precise treatment in CC. **l**–**n** Correlation of the RFS-related risk score and expression level of targets of precise treatment in CC

We identified the diagnostic application of risk signatures in the combined data using ROC curve analysis. The AUC for *ATG4C*, *ATG4D*, *CD46*, *HSPB8*, *MTMR14* and *SUPT20H* were 0.704, 0.640, 0.697, 0.627, 0.743 and 0.705 respectively (95% CI 0.612–0.796, 0.542–0.737, 0.603–0.791, 0.528–0.726, 0.654–0.832, 0.614–0.796), (Fig. 6a-f). This demonstrated that risk signatures were potential diagnostic markers. Diagnostic values of other risk signatures without statistical significance were shown in Additional file 7: Figure S5a-f.

### Single-gene Gene Set Enrichment for the risk signatures

Single-gene GSEA of the risk signatures has shown the potential roles of the genes in CC. The visualized results were filtered with *P* < 0.05 and FDR < 0.25, as shown in Additional file 7: Figure S6a–k. These genes were enriched in six different signal pathways, namely KEGG_ REGULATION_OF_AUTOPHAGY (*ATG4C*, *ATG4D*, *KLHL24*, *TP73*), KEGG_ MTOR_SIGNALING_PATHWAY (*HGS*, *VAMP7*), KEGG_NEUROTROPHIN_SIGNALING_PATHWAY (*KLHL24*), KEGG_PROSTATE_CANCER (*SERPINA1*, *SUPT20H*), KEGG_PROSTATE_CANCER (*SUPT20H*) and KEGG_FOCAL_ADHESION (*SUPT20H*). Details were shown in Additional file 6.

### Risk score could be applied as a sensitive indicator of target therapy

Pearson correlation analysis manifested our OS-related risk score was significantly related to the mRNA expression level of *AKT1*(cor = 0.1565, *P* = 0.0075), *BCL2* (cor = − 0.2589, *P* < 0.0001), *MTOR* (cor = 0.2013, *P* = 0.0006), *TP53* (cor = 0.2013, *P* = 0.0006), *VEGFA* (cor = − 0.3215, *P* < 0.0001) and RFS-related risk score was correlated with the mRNA expression level of *AKT1* (cor = 0.2630, *P* = 0.0002), *TP53* (cor = 0.1744, *P* = 0.0130), *VEGFA* (cor = 0.2093, *P* = 0. 0028), as shown in Fig. 6g–n. These results pointed out that patients with higher OS-related risk score and RFS-related risk score might respond better to therapies targeting *AKT1* and *VEGFA*, patients with lower OS-related risk score might respond better to therapies targeting *BCL2*. Besides, patients with higher OS-related risk score might be more sensitive to drugs targeting *MTOR*. Interestingly, patients with higher OS-related risk score and RFS-related risk score had opposite sensitivity to *TP53*, which was worth exploring further.

### RNA expression in CC and normal samples

**Fig. 7:**
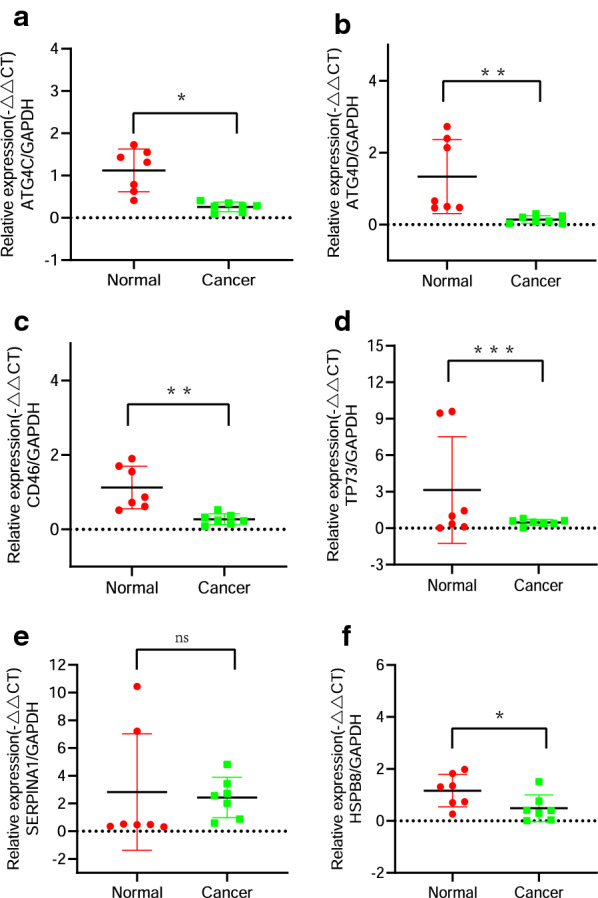
RNA expression of *ATG4C*, *ATG4D*, *CD46*, *TP73*, *SERPINA1* and *HSPB8* in CC and normal samples. **a**–**f** qPCR determined the RNA expression of *ATG4C*, *ATG4D*, *CD46*, *TP73*, *SERPINA1* and *HSPB8* in CC and normal samples. Quantitative normalization of the gene was performed in each sample using GAPDH expression as an internal control

Finally, among the 12 autophagy biomarkers related to the survival of cervical cancer, based on literature search, we selected 6 genes (*ATG4C*, *ATG4D*, *CD46*, *TP73*, *SERPINA1* and *HSPB8*) to verify their RNA expression in CC and normal cervical tissue samples. The results showed that except for *SERPINA1*, the other five genes were down-regulated in cervical cancer(Fig. 7a–f, all *P* < 0.05). This result shows that *ATG4C*, *ATG4D*, *CD46*, *TP73* and *HSPB8* may play a protective role in the progression of cervical cancer. Interestingly, *ATG4D*, *CD46*, *TP73* and *HSPB8* were negatively correlated with the risk score, which is consistent with the experimental results. *ATG4C* is positively correlated with the risk score, which is contrary to the above qPCR results.

## Discussion

CC is the most universal gynecological malignancy. In economically underdeveloped countries, the incidence and mortality of CC remain high all year round. 2018 International Federation of Obstetrics and Gynecology (FIGO) CC patient staging system proposed for the first time that imaging and pathological examination results should be included in the staging standard, which could reduce unnecessary surgery and the risk of complications in patients [21], but it had obvious limitations for patients with lymph node metastasis. Recently prognostic models of different cancer patients including CC have been constructed based on bioinformatics technology [14, 22–24]. It has been reported autophagy has a significant impact on the occurrence and development of tumors, autophagy-related genes can serve as prognostic markers [25–27]. However, few studies have reported the predictive relationship of CC transcriptome expression data on survival at the level of autophagy.

Inspired by the reported cellular and molecular effects of autophagy in CC, here we adopted autophagy-related genes expressed in CC rather than differentially expressed autophagy-related genes to construct prognostic models. After Cox regression analysis, we finally found nine autophagy-related risk signatures (*VAMP7*, *MTMR14*, *ATG4D*, *KLHL24*, *TP73*, *NAMPT*, *CD46*, *HGS*, *ATG4C*) were related to OS, and three autophagy-related risk signatures (*SERPINA1*, *HSPB8*, *SUPT20H*) were related to RFS. K-M survival analysis and the AUC illustrated our models had a great predictive performance. Additionally, the risk scores of the OS and RFS model could be considered as independent predictive indicators. Finally, the nomogram and calibration chart showed that the risk signatures could accurately assess the survival of CC patients. Vesicle-Associated Membrane Protein 7 (*VAMP7*) is a member of the synaptobrevin family which was found to be highly expressed and associated with a shorter survival period in esophageal cancer which could act as a dangerous gene [28]. However, we found out that *VAMP7* and risk score were negatively correlated contrary to previous conclusions. Myotubularin-related protein 14 (*MTMR14*) is a member of the myotubularin (MTM)-related protein family. Knockdown of *MTMR14* promoted cell apoptosis and inhibited migration in liver cancer cells [29]. However, Liu et al*.* put forward an opposite conclusion, they believed that the deficiency of *MTMR14* could promote autophagy and proliferation of mouse embryonic fibroblasts [30], which is consistent with our results. Autophagy Related 4D Cysteine Peptidase (*ATG4D*) and its paralog gene *ATG4C* played an important role as autophagy regulators that linked mitochondrial dysfunction with apoptosis in cancers such as breast carcinoma, human uterine fibroids, colorectal cancer and glioma [31–34]. Here we found that *ATG4C* might act as a risk factor, while *ATG4D* did the opposite. *KLHL24* has been uncovered as a prognostic predictor for acute myeloid leukemia [35]. Bockers suggested that *KLHL24* associated with *ESR1* could act as the upstream regulator to promote breast tumor growth [36]. In CC, high expression level of *KLHL24* can predict a better prognosis. *TP73* is a member of the *TP53* family whose expression has been observed altered in most human cancers and associated with the prognosis. In our present study, *TP73* played a protective role in the prognosis of CC. Nicotinamide phosphoribosyltransferase (*NAMPT*) possesses various functions in human cells, and altered *NAMPT* expression is associated with human carcinogenesis [37–39]. Currently, inhibition of *NAMPT* as a therapeutic strategy in cancer has attracted more and more attention. Our research once again confirmed the dangerous role of *NAMPT*. *CD46* has been found to be a prognostic indicator and highly expressed *CD46* was associated with poor prognosis [40, 41], which strongly supported our results. Hepatocyte Growth Factor-Regulated Tyrosine Kinase Substrate (*HGS*) is one of the master regulators whose expression gradually increased with CRC canceration and was an independent poor prognostic factor [42]. We also found that overexpressed *HGS* was associated with poor prognosis in CC. Serpin Family A Member 1 (*SERPINA1*) is a protein with a highly conserved structure and the most important protease inhibitor in humans whose expression is increased in malignant tumors, and it has been proven to be a predictor of poor prognosis for high-grade gliomas (HGGs), cutaneous squamous cell carcinoma (SCC) and non-small-cell lung cancer (NSCLC) [43–45]. Contrary to the above conclusion, *SERPINA1* played an inhibitory role in the poor prognosis of CC, which suggested that different cancers had different mechanisms. Heat Shock Protein Family B (Small) Member 8 (*HSPB8*) belongs to the small heat shock protein(*HSP20*)family. It has been reported that high *HSPB8* levels have a worse prognosis than low *HSPB8* expression [46, 47]. Veyssiere et al*.* proposed that the mutation of *SPT20* Homolog, SAGA Complex Component (*SUPT20H*) was significantly related to Rheumatoid Arthritis (RA) [48]). Additionally, *SUPT20H* could serve as an independent prognostic biomarker in gliomas and multiple myeloma (MM) [49, 50].

Next, we selected six genes(*ATG4C*, *ATG4D*, *CD46*, *TP73*, *SERPINA1* and *HSPB8*) from the above twelve risk biomarkers for qPCR verification according to literature retrieval. The experimental results confirm that *ATG4D*, *CD46*, *TP73* and *HSPB8* are decreased in cervical cancer, and the prognosis model shows a negative correlation with the risk score, it could be inferred that they had a protective effect on the occurrence and prognosis of cervical cancer. *ATG4C* expression is decreased in cervical cancer, but it is a risk factor in the prognosis model, it is worthy of further study.

The gene expression information and clinical survival information of patients with endometrial cancer (TCGA-UCEC) were validated to determine the predictive value of the OS model. Similarly, head and neck cancer (GSE117973) was downloaded to identify the predictive value of the RFS model. The results of the external validation set showed that our risk score was significantly related to the survival of patients, and a high risk score could indicate a poor prognosis for patients. Although the type of cancer in the testing set was different from the training set, studies have shown radiotherapy for CC can increase the incidence of endometrial cancer [19]. One of the important pathogenic factors for head and neck cancer is HPV infection [51]. So we believed that the prediction models for CC patients could be applied for endometrial cancer and head and neck cancer. These results certificated that the models had wide applicability.

Bioinformatics functional analysis showed that 53 DE-ARGs were mainly enriched in autophagy and apoptosis pathways. Based on the results, we hypothesized that autophagy played an important role in the occurrence and development of CC. Single-gene GSEA revealed the risk signatures included in our computational prognostic models could participate in autophagy, cell adhesion to affect the cell cycle, cell proliferation and migration.

Finally, we further explored whether model genes could be regarded as diagnostic markers. Interestingly, ROC curve analysis revealed that the patient might be accurately distinguished from the normal samples according to the expression of the risk signatures. In the present study, prognostic markers and diagnostic signatures were combined in CC for the first time, which explained that the incidence and prognosis of cancer were continuous and indivisible. Additionally, OS-related risk score and RFS-related risk score could prompt the patient’s sensitivity to the target drug to guide reasonable clinical treatment.

## Conclusion

In summary, we have constructed predictive models to predict the OS and RFS of CC patients based on ARGs expressed in CC and verified the effectiveness of the models in external validation sets. Furthermore, these model genes are expected to be diagnostic and treatment indicators, which may require a larger sample size to confirm this finding.

## Supplementary Information

**Additional file 1.** 232 autophagy-related genes from HADb.

**Additional file 2.** 217 autophagy-related genes expressed in CC.

**Additional file 3.** Primer sequence for qPCR.

**Additional file 4.** 53 differently expressed ARGs (DEARGs) in CC.

**Additional file 5.** Significant top 8 GO annotation and top 10 KEGG pathway enrichment.

**Additional file 6.** Detailed results of GSEA enrichment analysis.

**Additional file 7: Figure S1.** The box plot of 53 DEARGs and barplot of PPI network. **a.** The expression pattern of DEARGs in CC and normal samples. **b.** The number of nodes in the top 30 autophagy-related genes of the PPI network. **Figure S2.** Immunohistochemistry (IHC) results showing protein levels of autophagy-related genes in CC and normal tissues. **a.** IHC results of *ATG4C* in CC (staining:Medium; intensity:Moderate; quantity: > 75%; location: Cytoplasmic membranous) and in normal tissue (staining:Low;intensity:Weak; quantity:75%-25%; location:Cytoplasmic membranous).**b.** IHC results of *ATG4D* in CC (staining:Not detected; intensity:Negative; quantity:None; location:None) and in normal tissue(staining: Low;intensity:Weak; quantity:75%-25%; location:Cytoplasmic membranous). **c.** IHC results of *CD46* in CC(staining:Medium; intensity:Moderate; quantity: > 75%; location:Cytoplasmic membranous) and in normal tissue(staining:Low; intensity:Weak; quantity: > 75%; location: Cytoplasmic membranous). **d.** IHC results of *HGS* in CC(staining:Not detected; intensity:Negative; quantity:None; location:None) and in normal tissue(staining:Not detected; intensity:Negative; quantity:None; location:None). **e.** IHC results of *HSPB8* in CC(staining: Medium; intensity:Moderate; quantity: > 75%; location:Cytoplasmic membranous) and in normal tissue(staining:High; intensity:Strong; quantity: > 75%; location:Cytoplasmic membranous). **f.** IHC results of *KLHL24* in CC(staining:Not detected; intensity:Negative; quantity:None; location:None) and in normal tissue(staining:Not detected; intensity:Negative; quantity:None; location:None). **g.** IHC results of *MTMR14* in CC(staining:Not detected; intensity:Negative; quantity:None; location: None) and in normal tissue(staining:Not detected; intensity:Negative; quantity:None; location: None). **h.** IHC results of *NAMPT* in CC(staining:Not detected; intensity:Negative; quantity:None; location:None) and in normal tissue(staining:Not detected; intensity:Negative; quantity:None; location:None). **i.** IHC results of *SERPINA1* in CC(staining:Not detected; intensity:Negative; quantity:None; location:None) and in normal tissue(staining:Not detected; intensity:Negative; quantity:None; location:None). **j.** IHC results of *SUPT20H* in CC(staining:Medium; intensity:Moderate; quantity: > 75%; location:Cytoplasmic membranous) and in normal tissue(staining:Medium; intensity:Moderate; quantity: > 75%; location:Cytoplasmic membranous). **k.** IHC results of *TP73* in CC(staining:Not detected; intensity:Negative; quantity:None; location: None) and in normal tissue(staining:Medium;intensity:Strong; quantity: < 25%;location:Nuclear). **l.** IHC results of *VAMP7* in CC(staining:Medium; intensity:Moderate; quantity: > 75%; location: Cytoplasmic membranous) and in normal tissue(staining:Medium; intensity:Moderate; quantity: 75%-25%; location:Cytoplasmic membranous). **Figure S3.** Kaplan–Meier survival curves for CC based on the prognostic risk genes. **a-l** shows the results of *ATG4C*, *ATG4D*, *CD46*, *HGS*, *KLHL24*, *MTMR14*, *NAMPT*, *TP73*, *VAMP7*, *HSPB8*, *SERPINA1* and *SUPT20H* respectively. **Figure S4.** Calibration curve of nomogram diagram and exploration of risk signature in the testing set. **a-c.** Calibration plots for 1-year, 3-year and 5-year survival for the nomogram of OS model. **d-f.** Calibration plots for 1-year, 3-year and 5-year survival for the nomogram of RFS model. **g.** K-M curve of the high- and low-risk UCEC patients in the testing group for OS model. **h.** The 3-year, 5-year and 7-year ROC curves in the testing group of UCEC patients for OS. **i.** K-M curve of the high- and low-risk HNSCC patients in the testing group for the RFS model. **j.** The 1-year, 3-year and 5-year ROC curves in the testing group of HNSCC patients for RFS. **Figure S5.** AUC of risk signature without diagnostic value. **a-f.** AUC of risk signature without diagnostic value (*HGS*, *KLHL24*, *NAMPT*, *SERPINA1*, *TP73*, *NAMP7*). **Figure S6.** Single gene function enrichment analysis and target sensitivity detection. **a-k.** Single gene function enrichment analysis of 12 prognostic risk signatures.

## Data Availability

The datasets analysed during the current study are available from the corresponding author Shizhi Wang (shizhiwang2009@seu.edu.cn) on reasonable request. The data that support the findings of this study are available at the TCGA data portal (https://tcga-data.nci.nih.gov/tcga/), the GTEx portal (http://gtexportal.org) and the comprehensive Gene Expression Omnibus (GEO;https://www.ncbi.nlm.nih.gov/geo/).

## Abbreviations

AUC: The area under the curve
ARGs: Autophagy-related genes
BP: Biological process
CC: Cellular component
CC: Cervical cancer
CIN: Cervical intraepithelial neoplasia
DEARGs: Differently expressed autophagy-related genes
UCEC: Endometrial cancer
FIGO: Federation of Obstetrics and Gynecology
GEO: Gene Expression Omnibus
GO: Gene Ontology
KEGG: Kyoto Encyclopedia of Genes and Genomes
GSEA: Gene Set Enrichment Analysis
GTEx: Genotype-Tissue Expression
HNSCC: Head and neck cancer
HADb: Human Autophagy-dedicated Database
HPV: Human papillomavirus
HPA: Human Protein Atlas database
K-M curve: Kaplan–Meier curve
Lasso: Least absolute shrinkage and selection operator
MF: Molecular function
OS: Overall survival
PFS: Progression-free survival
PPI: Protein–protein interaction
ROC: Receiver operating characteristic
RFS: Recurrence-free survival
RNA-seq: RNA sequence
TCGA: The Cancer Genome Atlas
