# Supplementary figures and images for: Identification of autophagy-related risk signatures for the prognosis, diagnosis, and targeted therapy in cervical cancer
Source: Cancer Cell Int. 2021 Jul 8;21:362. doi: 10.1186/s12935-021-02073-w (PMC8268251; doi:10.1186/s12935-021-02073-w)

**a**

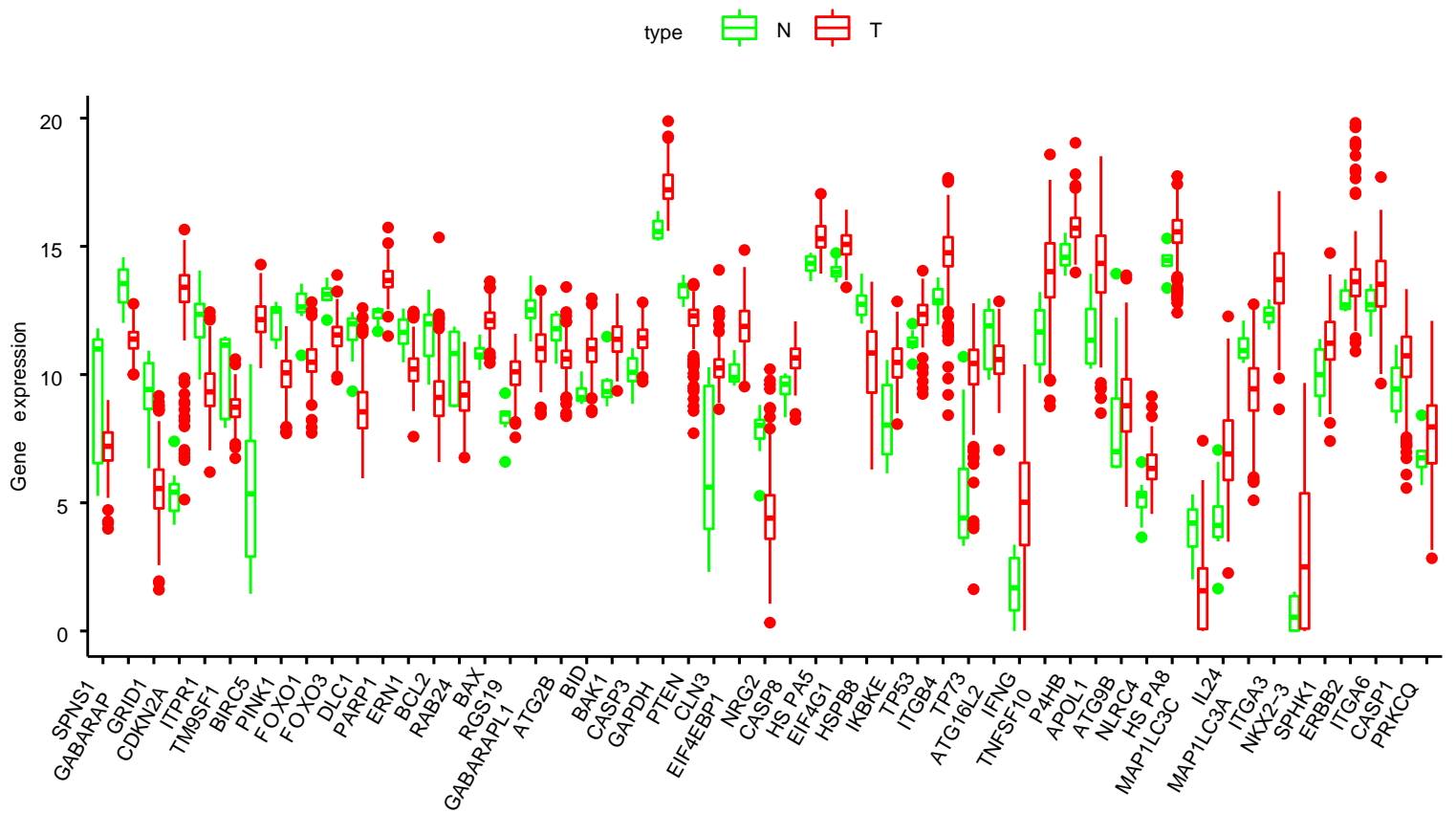

**b**

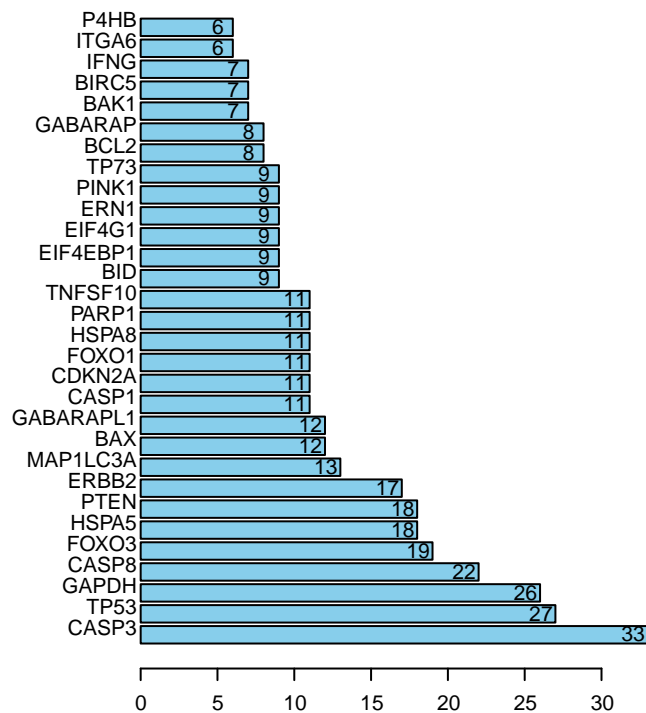

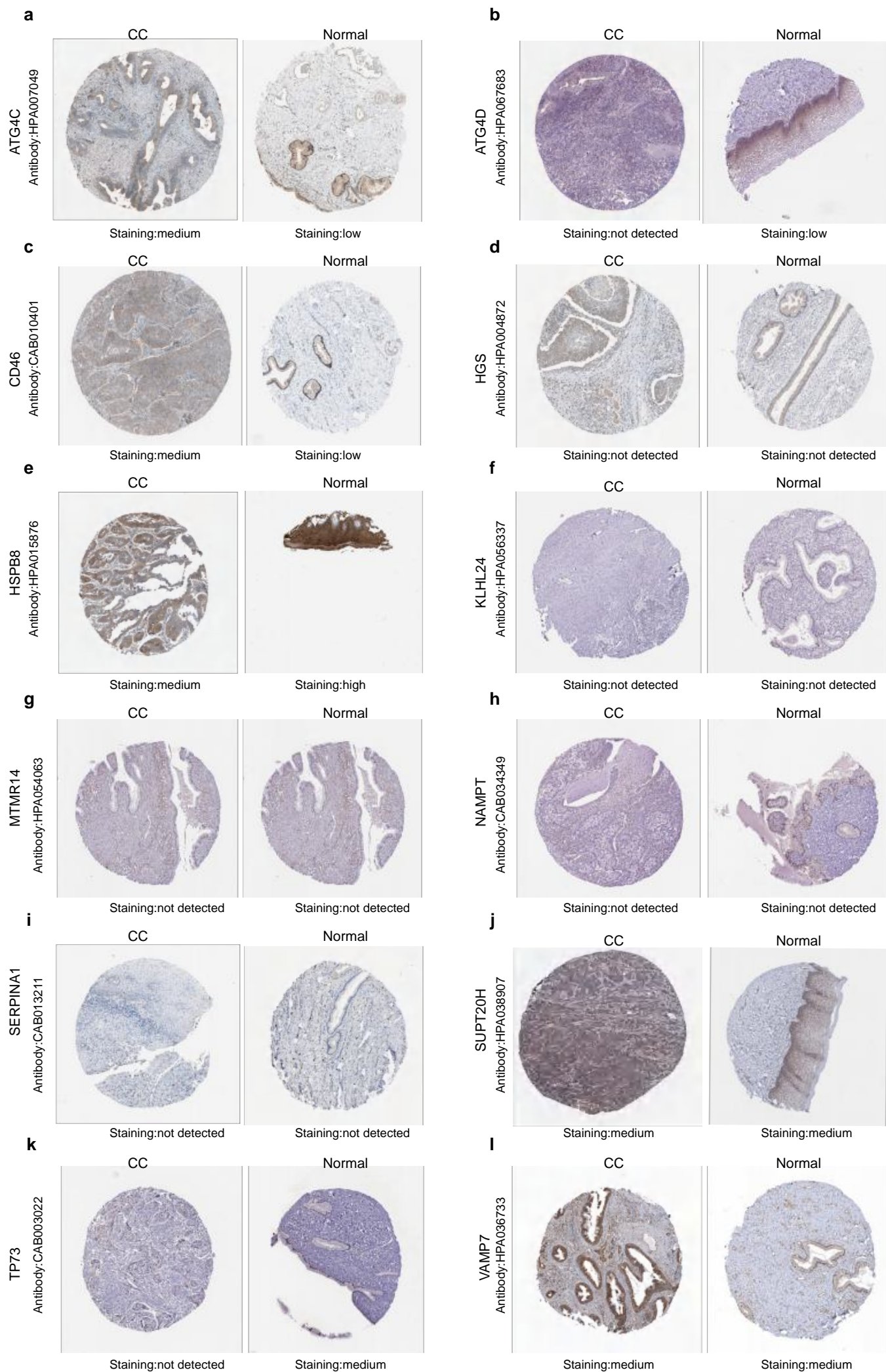

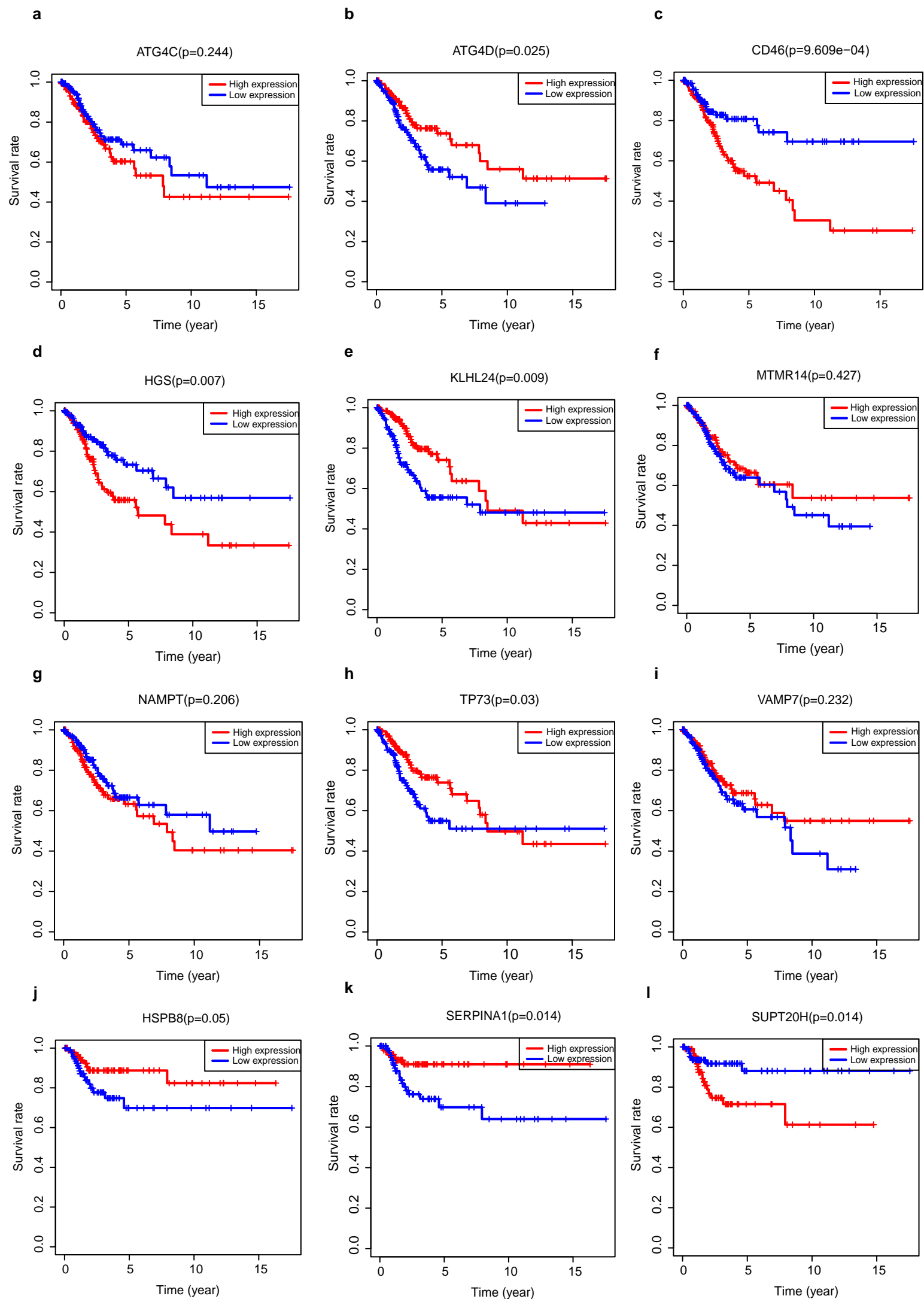

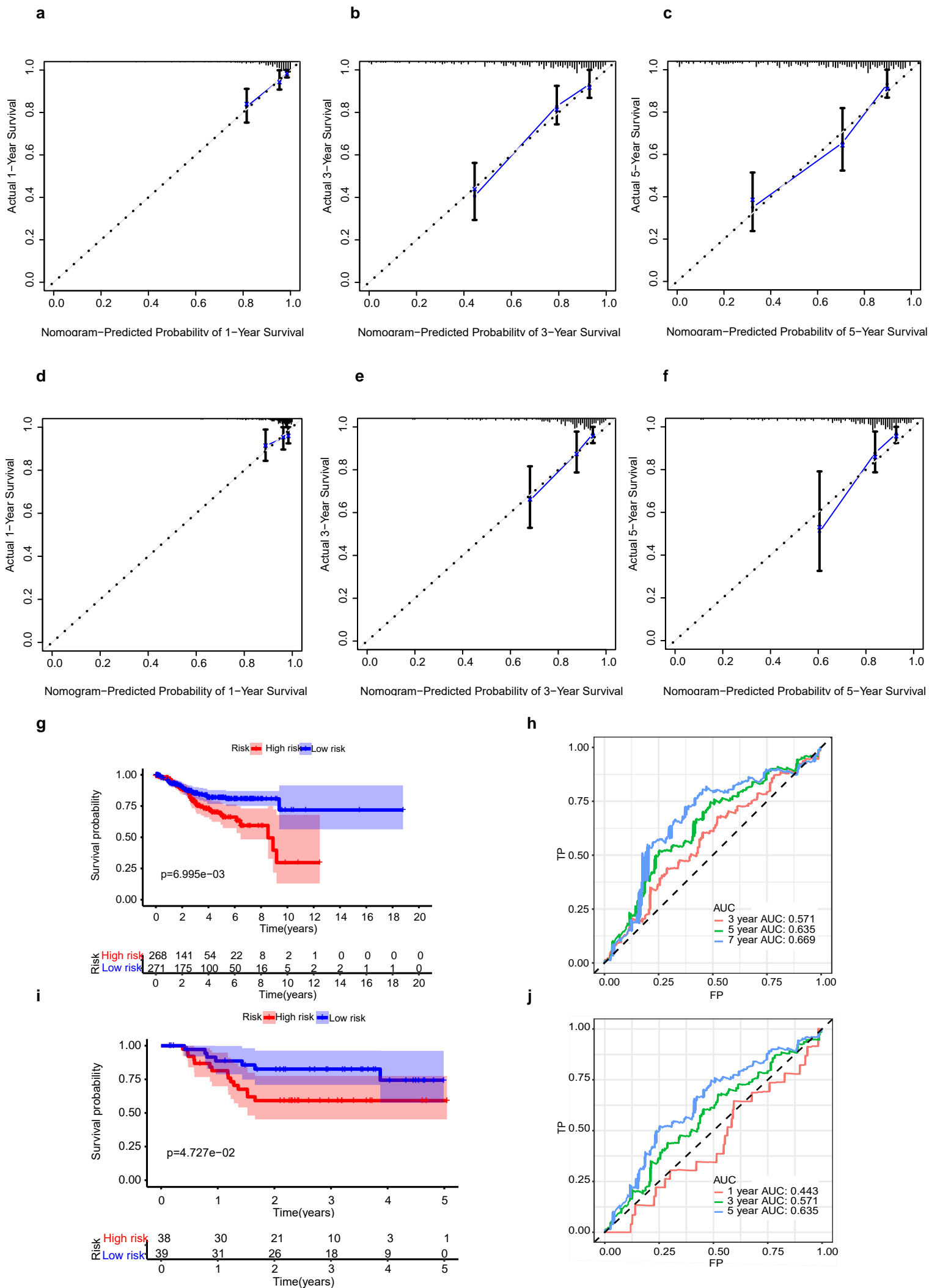

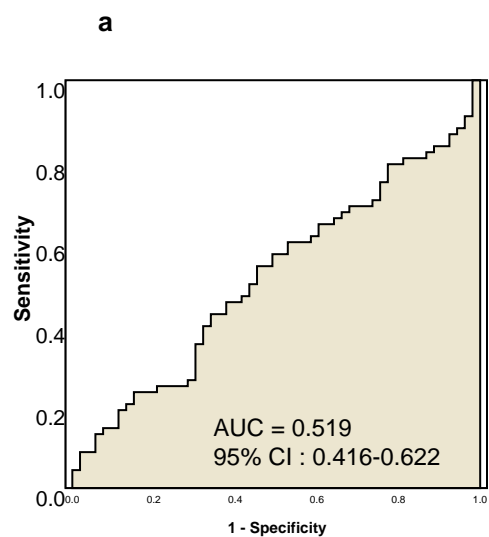

HGS

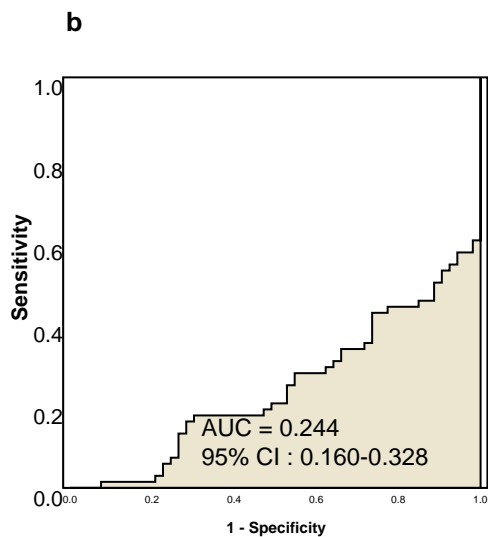

KLHL24

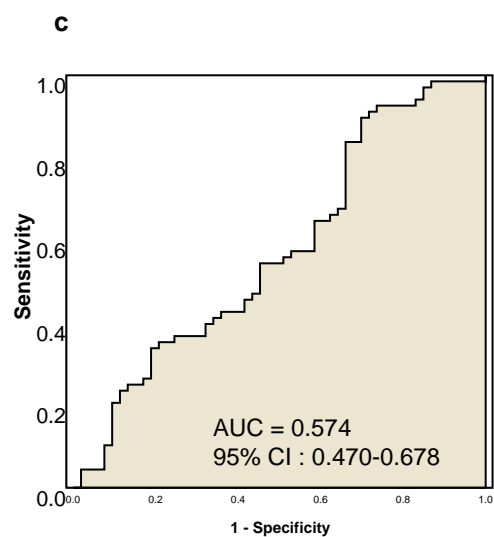

NAMPT

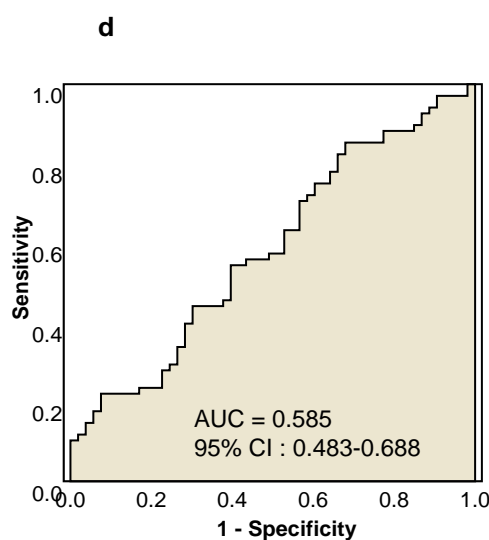

SERPINA1

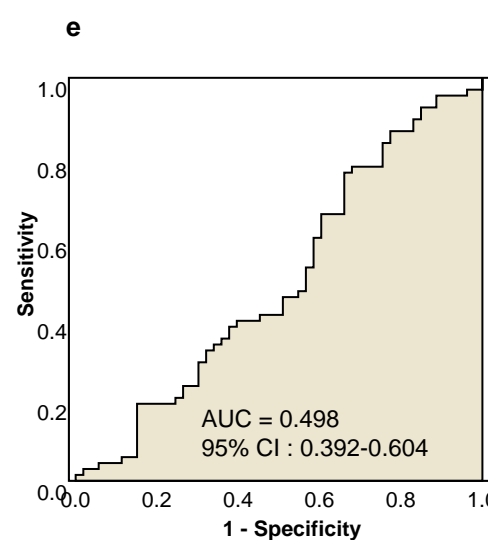

TP73

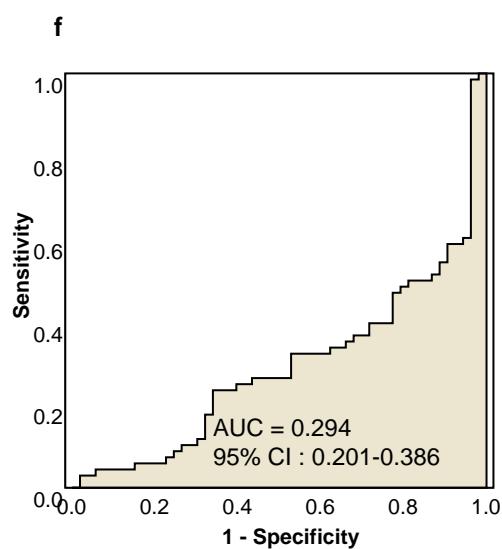

VAMP7

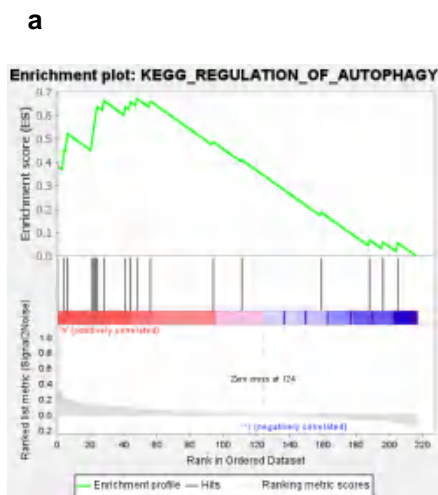

ATG4C

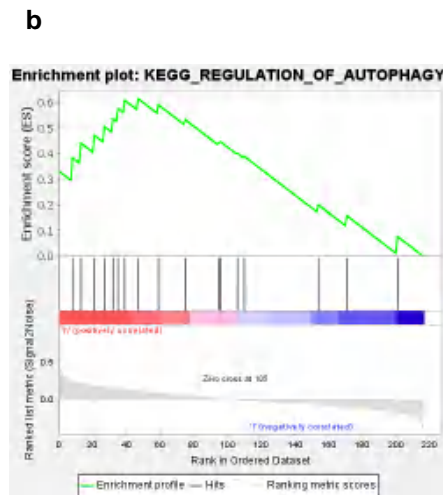

ATG4D

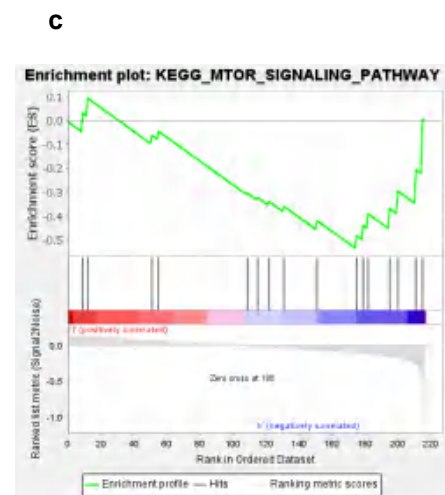

HGS

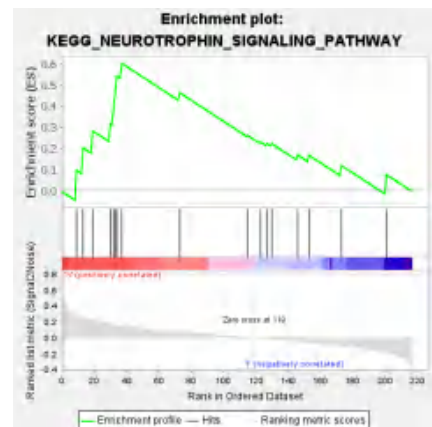

KLHL24

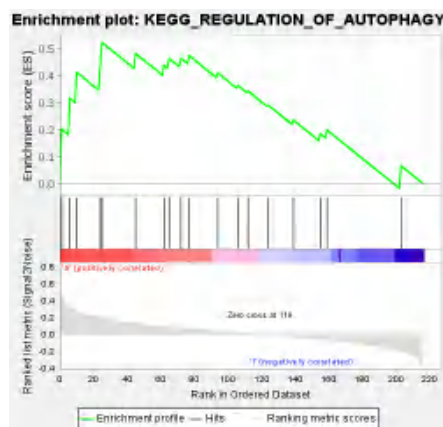

KLHL24

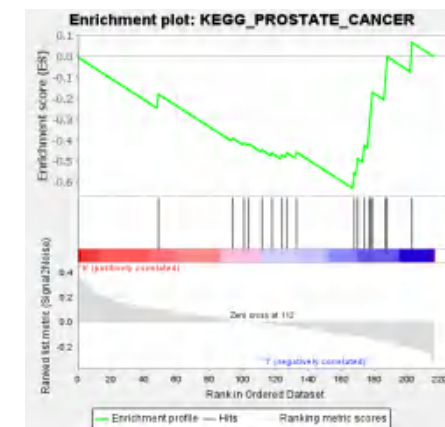

SERPINA1

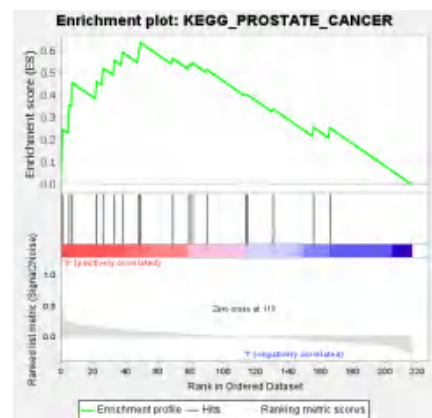

SUPT20H

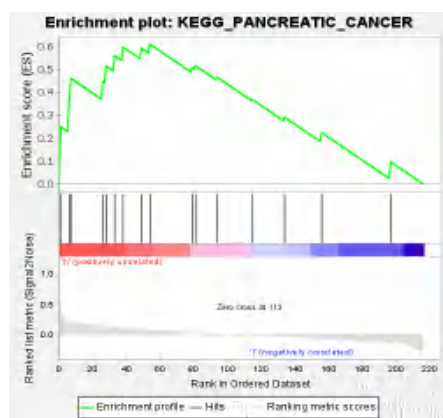

SUPT20H

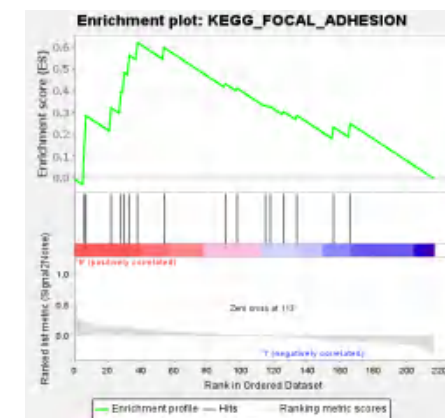

SUPT20H

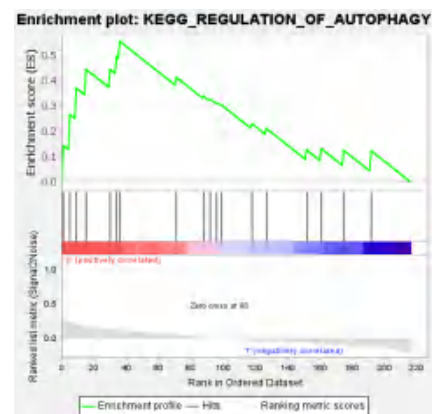

TP73

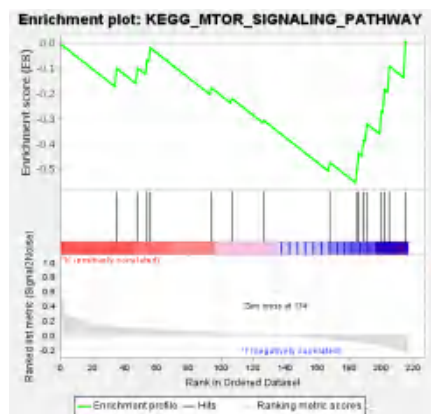

VAMP7

Supplement: Supplementary file 7 — Additional file 7: Figure S1. The box plot of 53 DEARGs and barplot of PPI network. a. The expression pattern of DEARGs in CC and normal samples. b. The number of nodes in the top 30 autophagy-related genes of the PPI network. Figure S2. Immunohistochemistry (IHC) results showing protein levels of autophagy-related genes in CC and normal tissues. a. IHC results of ATG4C in CC (staining:Medium; intensity:Moderate; quantity: > 75%; location: Cytoplasmic membranous) and in normal tissue (staining:Low;intensity:Weak; quantity:75%-25%; location:Cytoplasmic membranous).b. IHC results of ATG4D in CC (staining:Not detected; intensity:Negative; quantity:None; location:None) and in normal tissue(staining: Low;intensity:Weak; quantity:75%-25%; location:Cytoplasmic membranous). c. IHC results of CD46 in CC(staining:Medium; intensity:Moderate; quantity: > 75%; location:Cytoplasmic membranous) and in normal tissue(staining:Low; intensity:Weak; quantity: > 75%; location: Cytoplasmic membranous). d. IHC results of HGS in CC(staining:Not detected; intensity:Negative; quantity:None; location:None) and in normal tissue(staining:Not detected; intensity:Negative; quantity:None; location:None). e. IHC results of HSPB8 in CC(staining: Medium; intensity:Moderate; quantity: > 75%; location:Cytoplasmic membranous) and in normal tissue(staining:High; intensity:Strong; quantity: > 75%; location:Cytoplasmic membranous). f. IHC results of KLHL24 in CC(staining:Not detected; intensity:Negative; quantity:None; location:None) and in normal tissue(staining:Not detected; intensity:Negative; quantity:None; location:None). g. IHC results of MTMR14 in CC(staining:Not detected; intensity:Negative; quantity:None; location: None) and in normal tissue(staining:Not detected; intensity:Negative; quantity:None; location: None). h. IHC results of NAMPT in CC(staining:Not detected; intensity:Negative; quantity:None; location:None) and in normal tissue(staining:Not detected; intensity:Negative; quantity:None; loc [file 12935_2021_2073_MOESM7_ESM.pdf]
